# Supplementary material for: The Impact of Missing Data and Imputation Methods on the Analysis of 24-Hour Activity Patterns
Source: Clocks Sleep. 2022 Sep 27;4(4):497–507. doi: 10.3390/clockssleep4040039 (PMC9590093; doi:10.3390/clockssleep4040039)
Supplement: Supplementary file 1 [file clockssleep-04-00039-s001.zip › clockssleep-1873928-supplementary.pdf]

Supplementary Materials

The Impact of Missing Data and Imputation Methods on Calculation of 24-Hour Activity Patterns: Interdaily Stability and Intradaily Variability

Lara Weed, Renske Lok, Dwijen Chawra, and Jamie Zeitzer

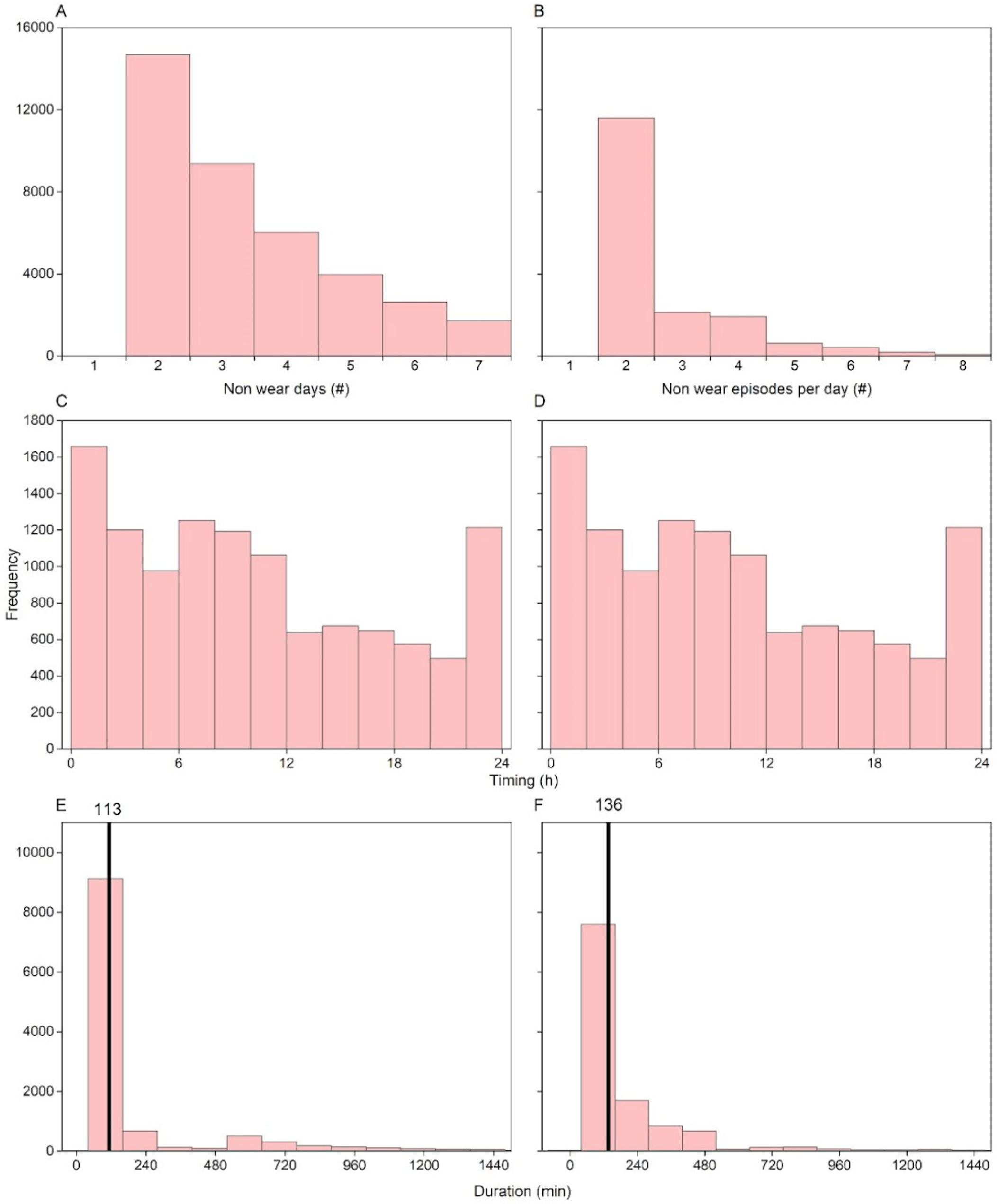

Figure S1. Histograms of non-wear episodes in the general population (n = 83,937). Indicated are the number of days that have non-wear episodes (A), the number of non-wear episodes per day (B), timing of the first (C) and second (D) episode of non-wear, and the duration of the first (E) and second (F) episode of missing data. Black lines indicate the geometric mean, which is 113 and 136 minutes for the first and second non-wear episode, respectively.

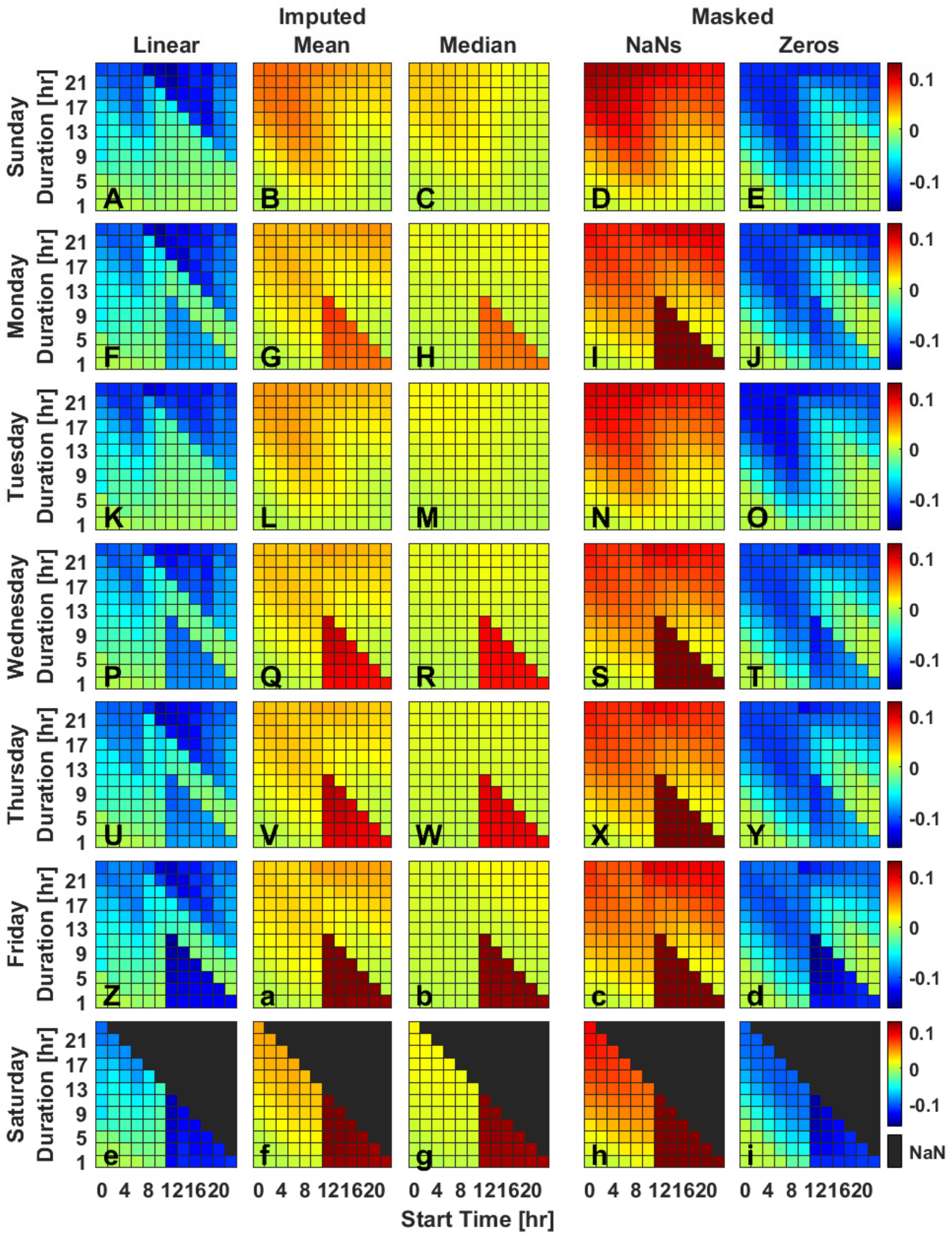

Figure S2. Interdaily Stability (IS) Bland-Altman mean for a single missing data gap with starting times across all days of the week. Data are the mean difference between the masked (i.e., Zeros and NaNs) and true IS values or imputed (i.e., Linear interpolation, Mean time of day (ToD), and Median ToD) and true IS values, as extracted from Bland-Altman plots. Values are presented for varied gap durations (y-axis) and timing (x-axis) of masked data gaps and are color-coded as indicated with best performance being closer to 0. NaN values indicate where values could not be calculated due to dataset constraints.

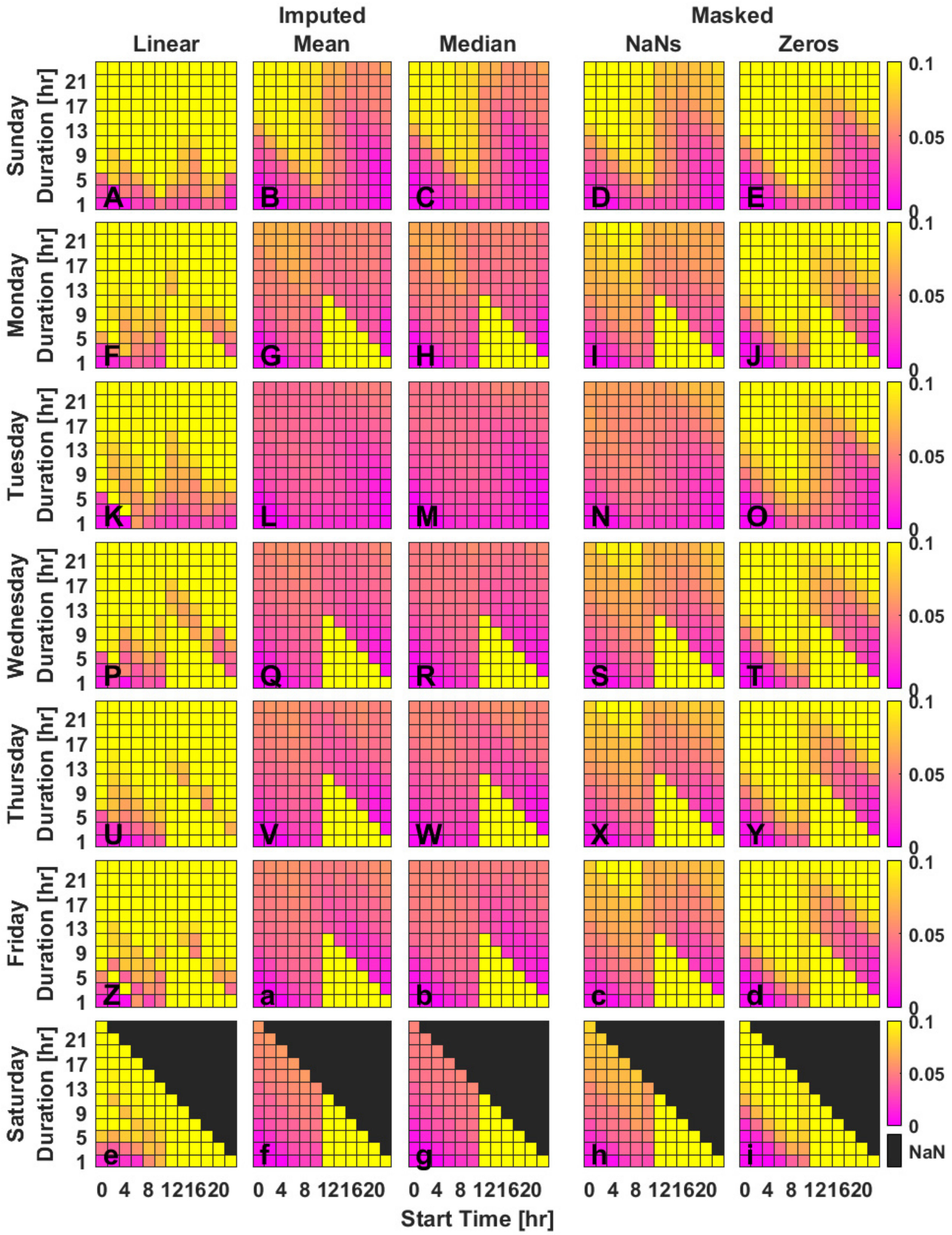

Figure S3. IS Bland-Altman 1.96*standard deviation for a single missing data gap with starting times across all days of the week. Data are the mean difference between the masked (i.e., Zeros and NaNs) and true IS values or imputed (i.e., Linear interpolation, Mean ToD, and Median ToD) and true IS values, as extracted from Bland-Altman plots. Values are presented for varied gap durations (y-axis) and timing (x-axis) of masked data gaps and are color-coded as indicated with best performance being closer to 0. NaN values indicate where values could not be calculated due to dataset constraints.

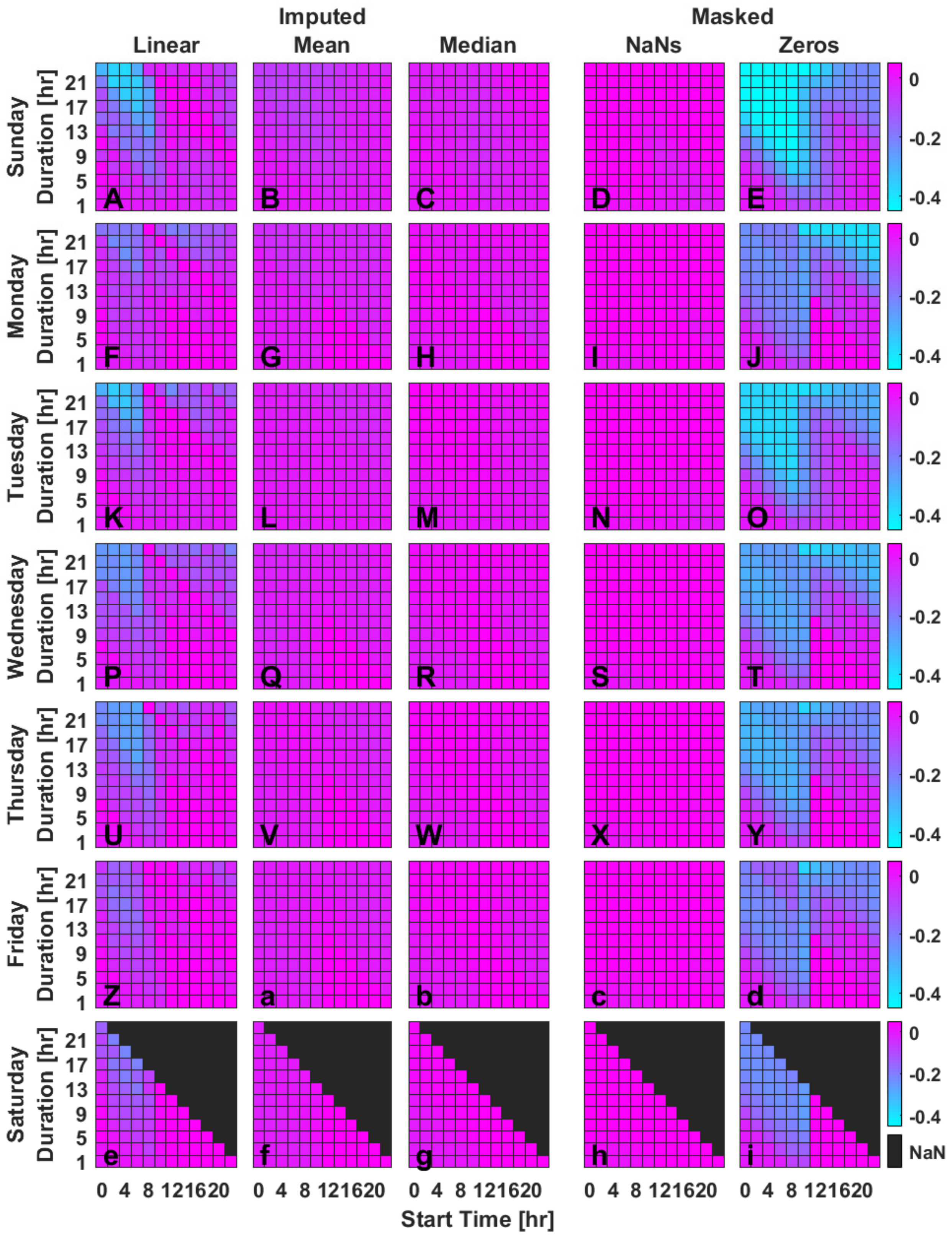

Figure S4. IS Bland-Altman slope for a single missing data gap with starting times across all days of the week. Data are the mean difference between the masked (i.e., Zeros and NaNs) and true IS values or imputed (i.e., Linear, Mean, and Median) and true IS values, as extracted from Bland-Altman plots. Values are presented for systematically varied gap durations (y-axis) and timing (x-axis) of masked data gaps and are color-coded as indicated with best performance being closer to 0. NaN values indicate where values could not be calculated due to dataset constraints.

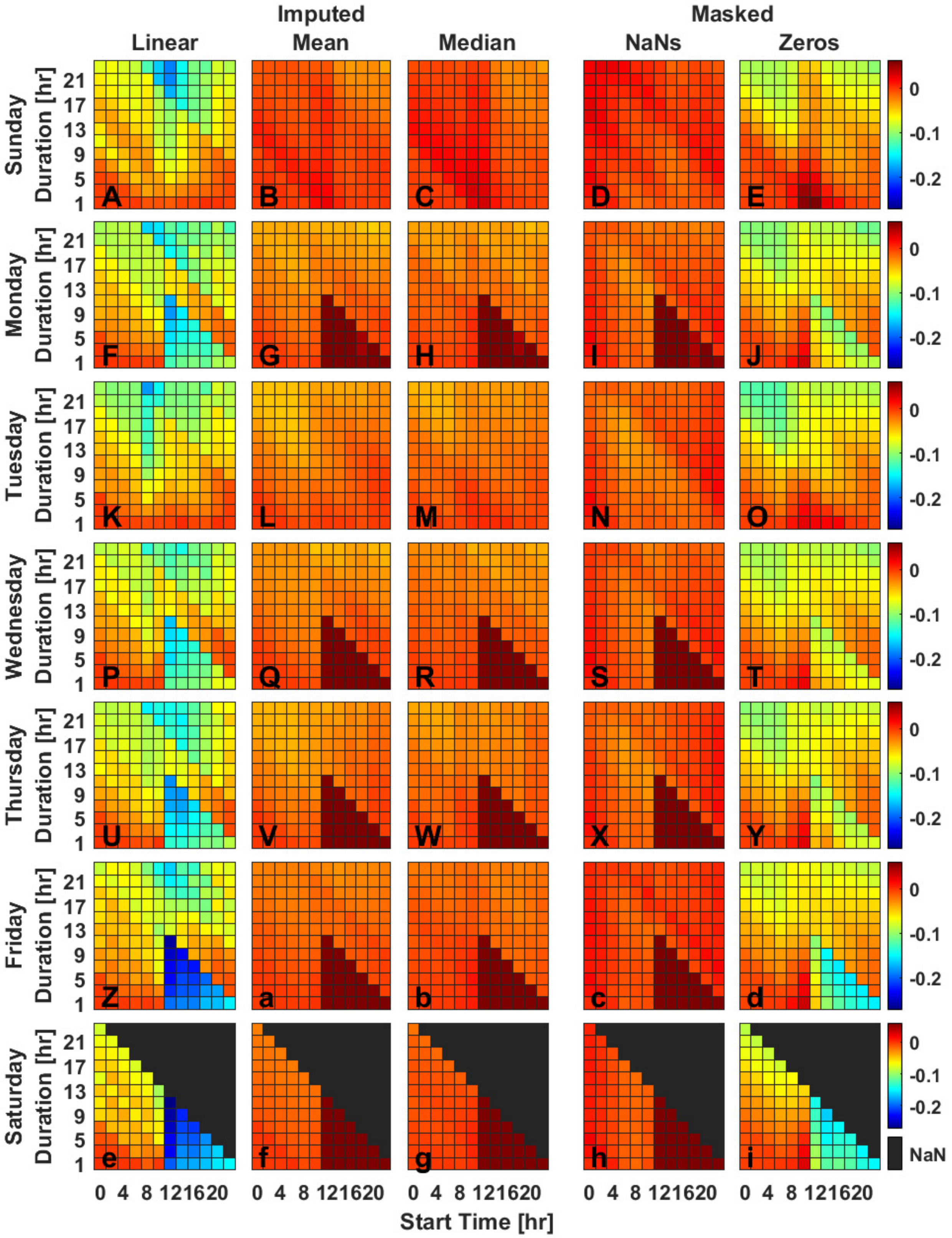

Figure S5. IV Bland-Altman mean for a single missing data gap with starting times across all days of the week. Data are the mean difference between the masked (i.e., Zeros and NaNs) and true IS values or imputed (i.e., Linear interpolation, Mean ToD, and Median ToD) and true IS values, as extracted from Bland-Altman plots. Values are presented for varied gap durations (y-axis) and timing (x-axis) of masked data gaps and are color-coded as indicated with best performance being closer to 0. NaN values indicate where values could not be calculated due to dataset constraints.

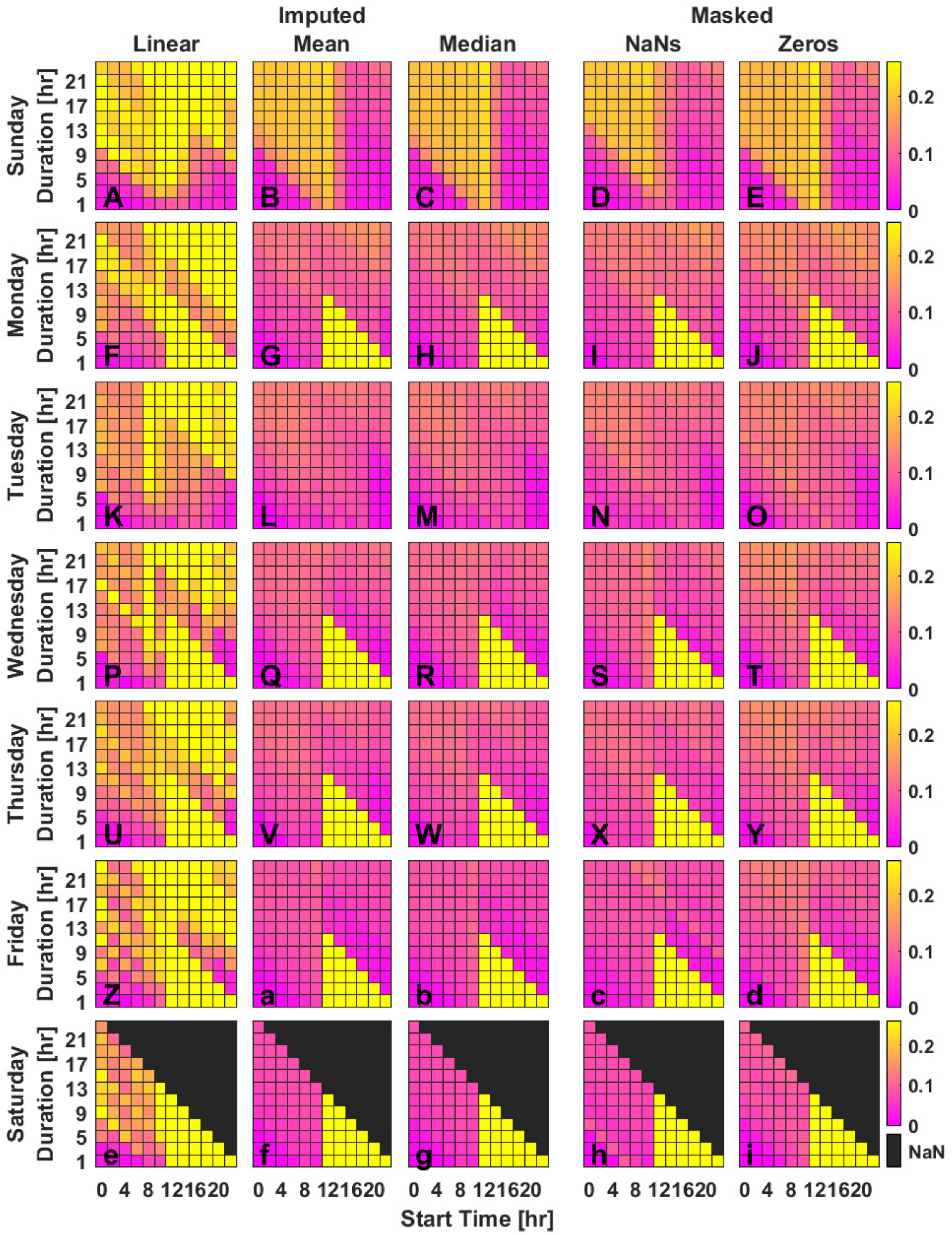

Figure S6. IV Bland-Altman 1.96*standard deviation for a single missing data gap with starting times across all days of the week. Data are the mean difference between the masked (i.e., Zeros and NaNs) and true IV values or imputed (i.e., Linear interpolation, Mean ToD, and Median ToD) and true IV values, as extracted from Bland-Altman plots. Values are presented for varied gap durations (y-axis) and timing (x-axis) of masked data gaps and are color-coded as indicated with best performance being closer to 0. NaN values indicate where values could not be calculated due to dataset constraints.

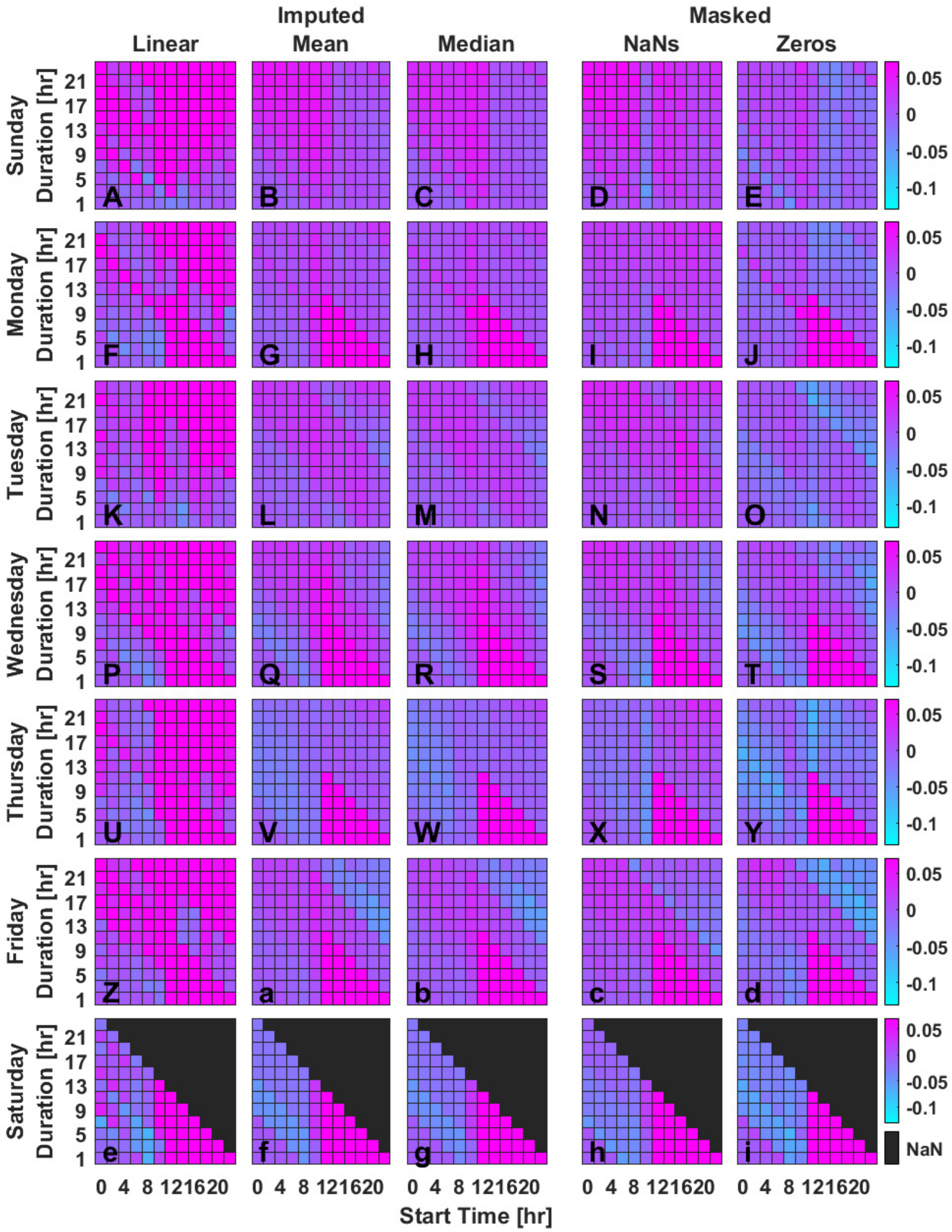

Figure S7. IV Bland-Altman slope for a single missing data gap with starting times across all days of the week. Data are the mean difference between the masked (i.e., Zeros and NaNs) and true IV values or imputed (i.e., Linear interpolation, Mean ToD, and Median ToD) and true IV values, as extracted from Bland-Altman plots. Values are presented for varied gap durations (y-axis) and timing (x-axis) of masked data gaps and are color-coded as indicated with best performance being closer to 0. NaN values indicate where values could not be calculated due to dataset constraints.

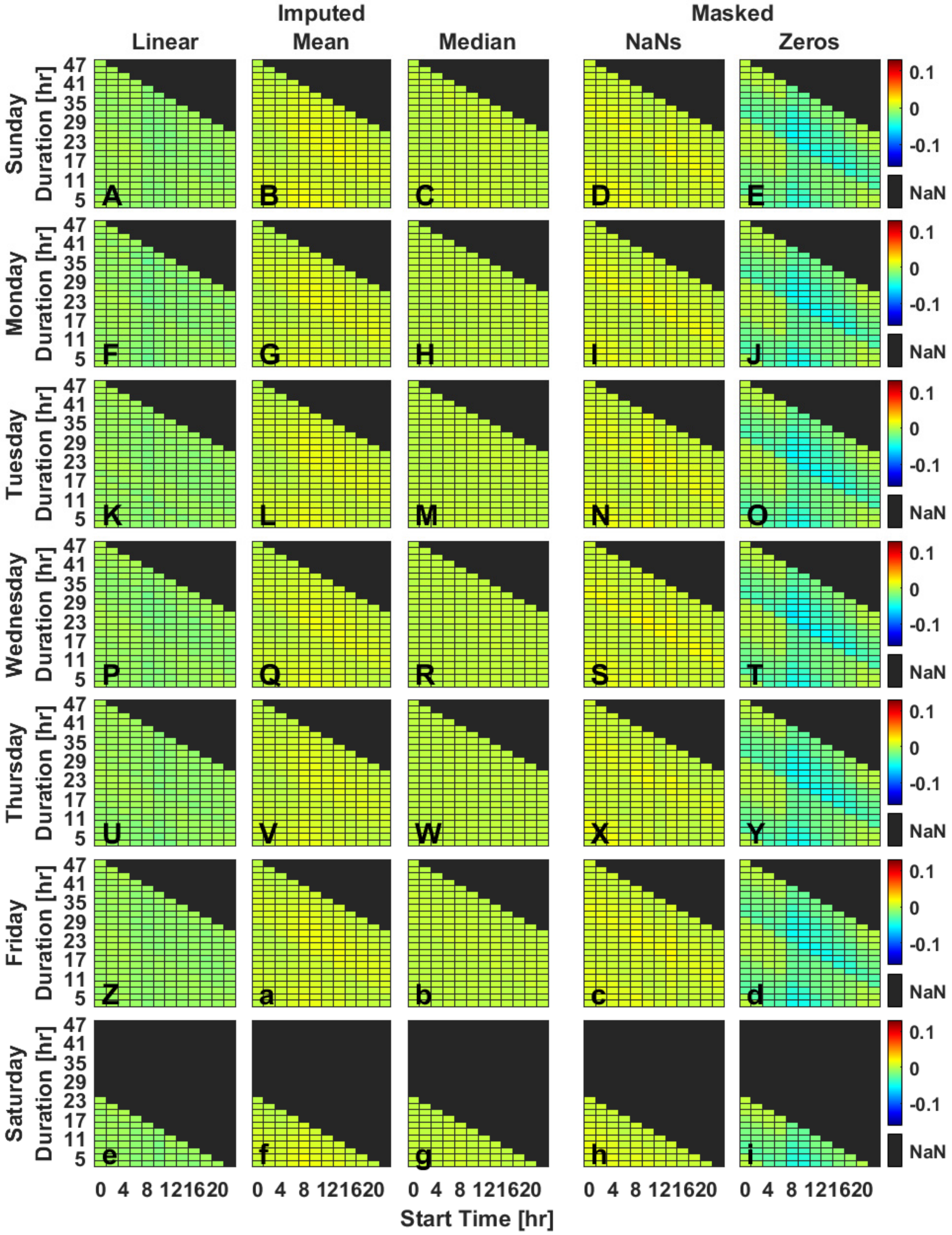

Figure S8. IS Bland-Altman mean for two gaps (gap 1: 115 minutes, gap 2: 140 minutes) of missing data with starting times across all days of the week. Data are the mean difference between the masked (i.e., Zeros and NaNs) and true IS values or imputed (i.e., Linear interpolation, Mean ToD, and Median ToD) and true IS values, as extracted from Bland-Altman plots. Values are presented for varied durations between bouts (y-axis) and timing (x-axis) of masked data gaps and are color-coded as indicated with best performance being closer to 0. NaN values indicate where values could not be calculated due to dataset constraints.

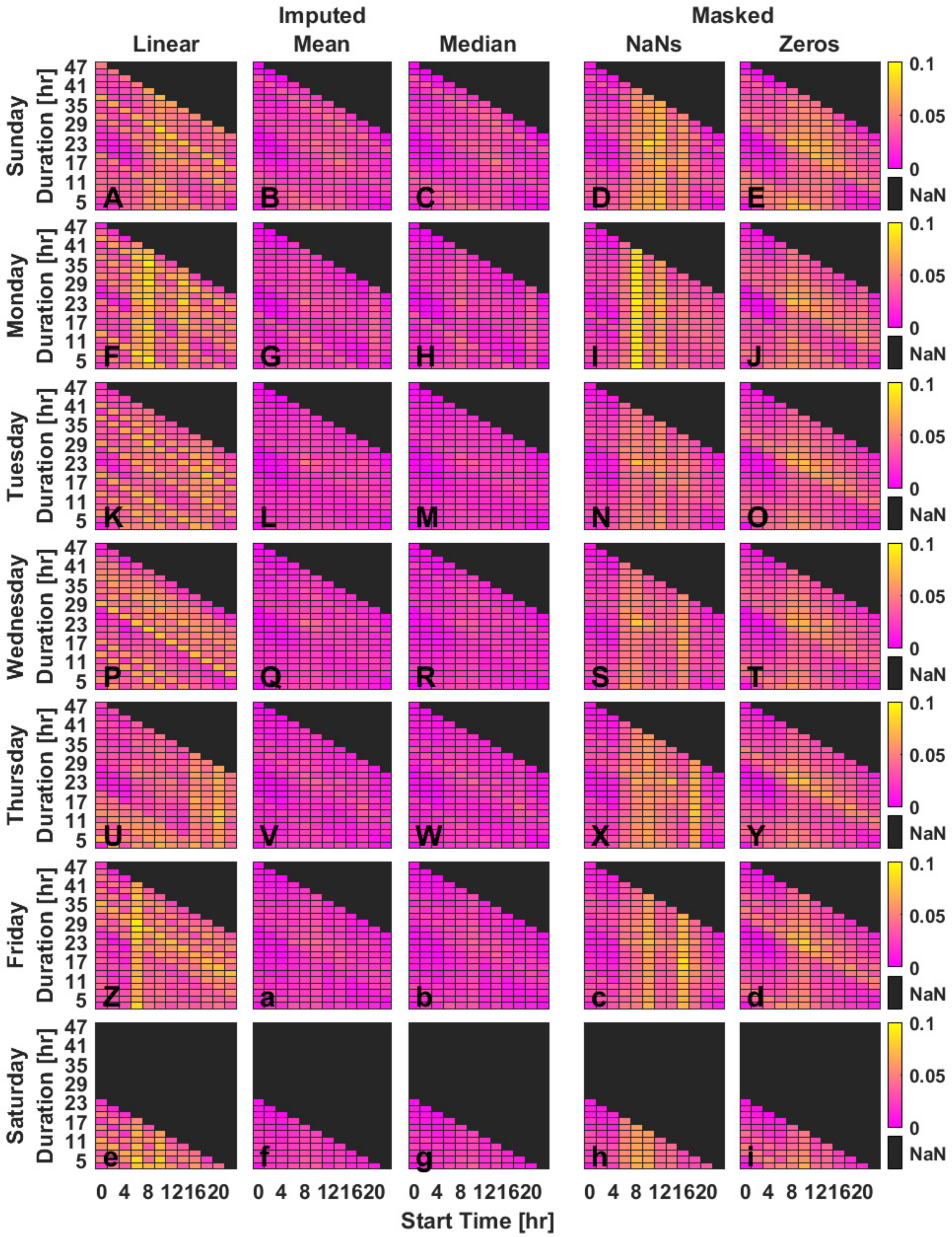

Figure S9. IS Bland-Altman 1.96*standard deviation for two gaps (gap 1: 115 minutes, gap 2: 140 minutes) of missing data with starting times across all days of the week. Data are the mean difference between the masked (i.e., Zeros and NaNs) and true IS values or imputed (i.e., Linear interpolation, Mean ToD, and Median ToD) and true IS values, as extracted from Bland-Altman plots. Values are presented for varied durations between bouts (y-axis) and timing (x-axis) of masked data gaps and are color-coded as indicated with best performance being closer to 0. NaN values indicate where values could not be calculated due to dataset constraints.

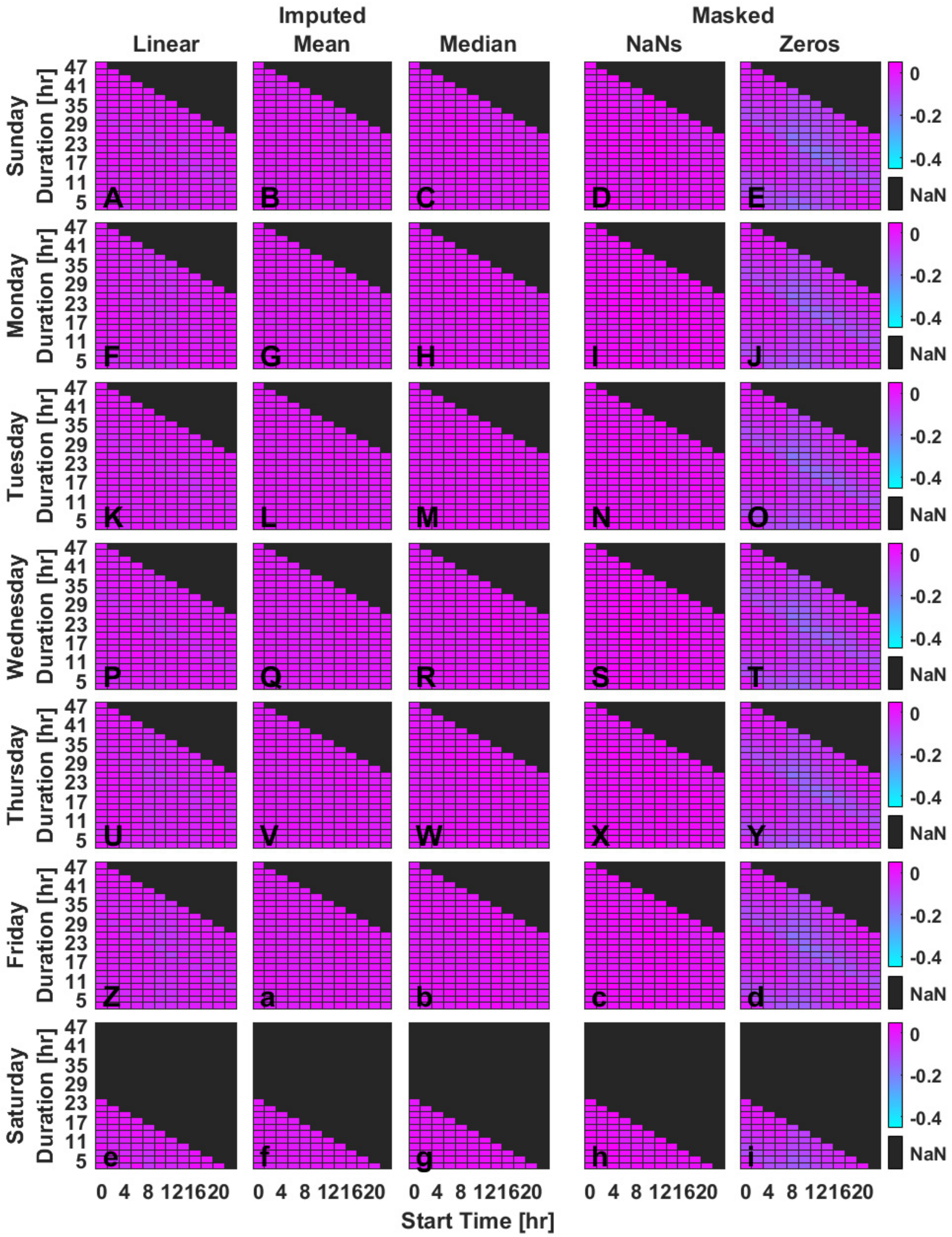

Figure S10. IS Bland-Altman slope for two gaps (gap 1: 115 minutes, gap 2: 140 minutes) of missing data with starting times across all days of the week. Data are the mean difference between the masked (i.e., Zeros and NaNs) and true IS values or imputed (i.e., Linear interpolation, Mean ToD, and Median ToD) and true IS values, as extracted from Bland-Altman plots. Values are presented for varied durations between bouts (y-axis) and timing (x-axis) of masked data gaps and are color-coded as indicated with best performance being closer to 0. NaN values indicate where values could not be calculated due to dataset constraints.

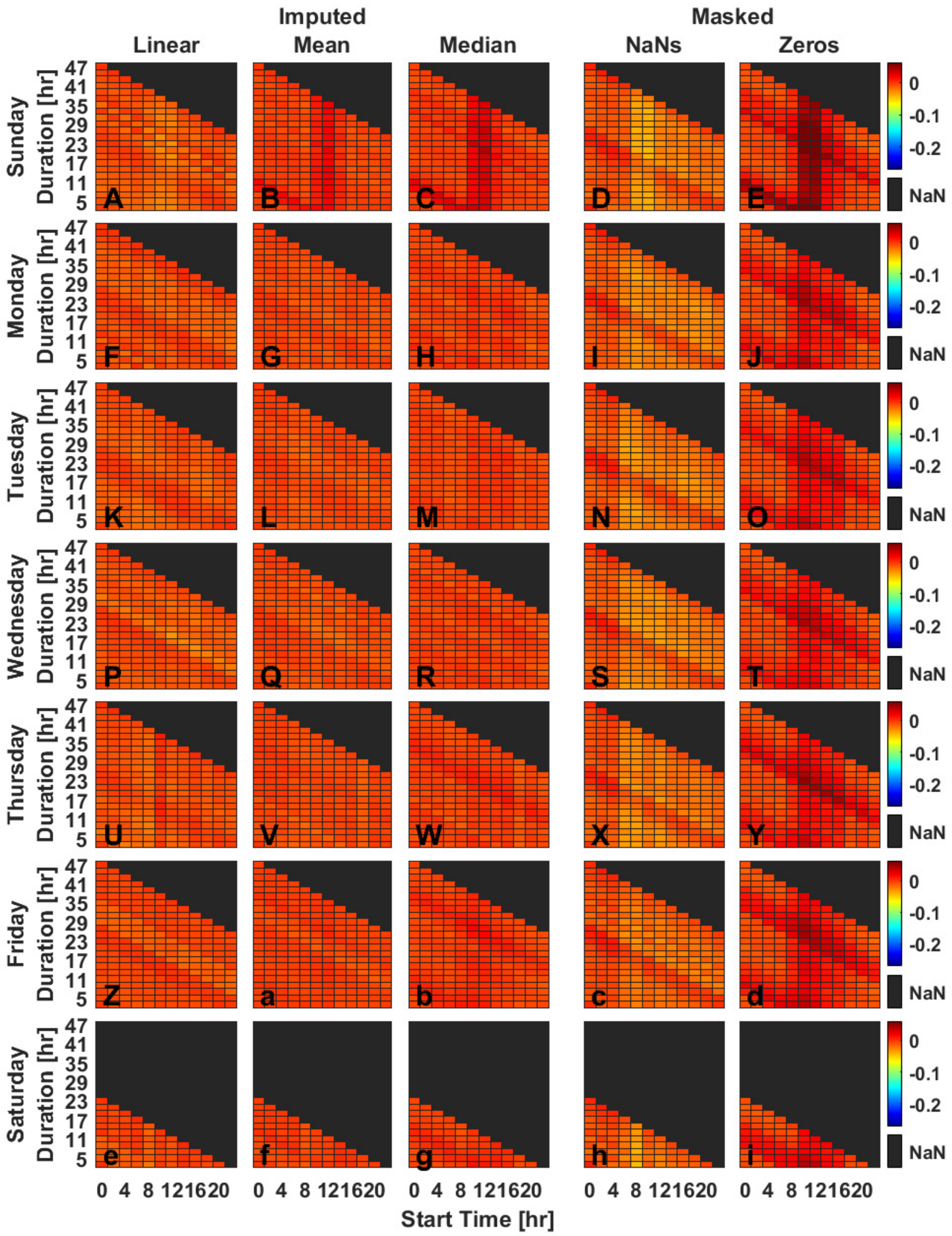

Figure S11. IV Bland-Altman mean for two gaps (gap 1: 115 minutes, gap 2: 140 minutes) of missing data with starting times across all days of the week. Data are the mean difference between the masked (i.e., Zeros and NaNs) and true IV values or imputed (i.e., Linear interpolation, Mean ToD, and Median ToD) and true IV values, as extracted from Bland-Altman plots. Values are presented for varied durations between bouts (y-axis) and timing (x-axis) of masked data gaps and are color-coded as indicated with best performance being closer to 0. NaN values indicate where values could not be calculated due to dataset constraints.

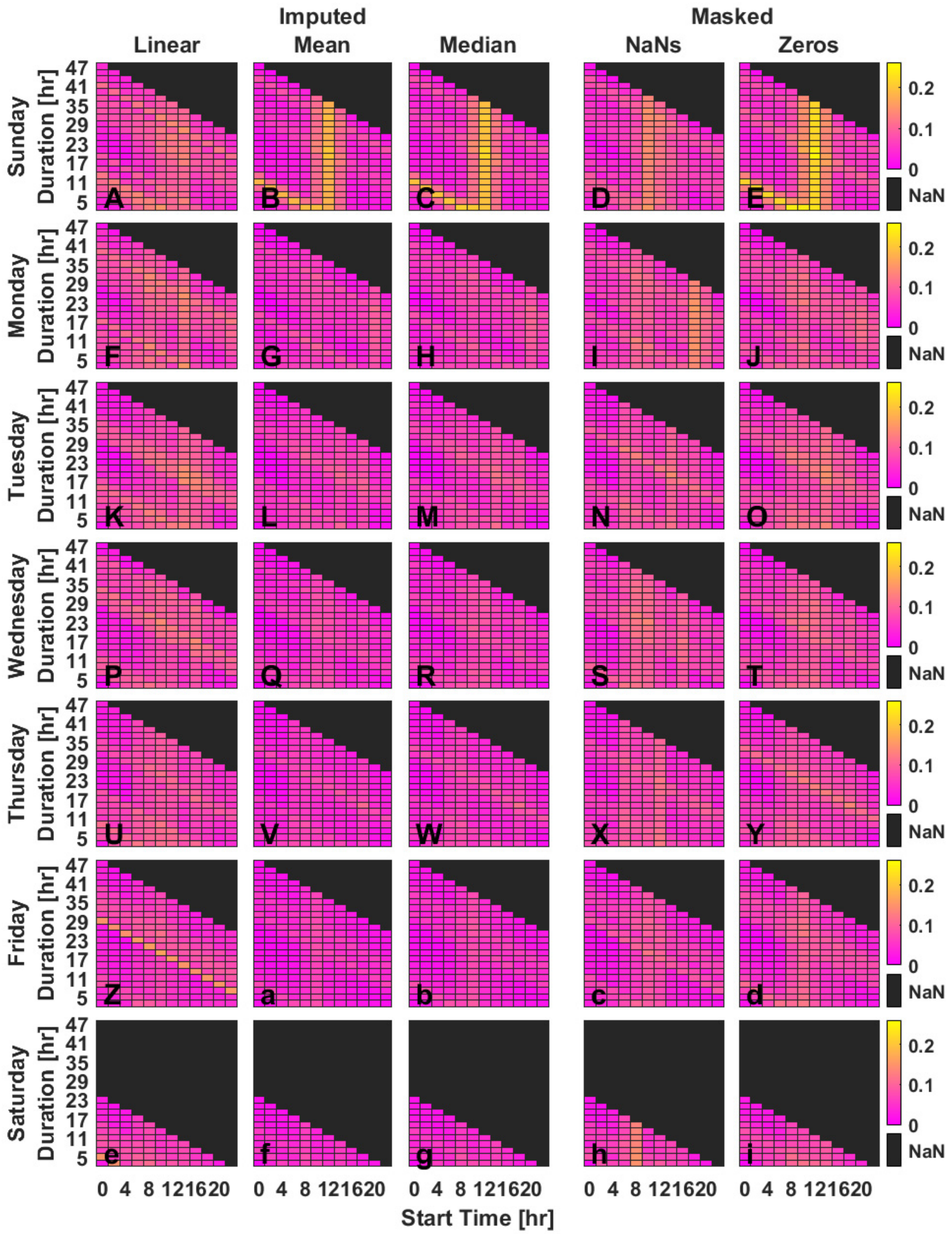

Figure S12. IV Bland-Altman 1.96*standard deviation for two gaps (gap 1: 115 minutes, gap 2: 140 minutes) of missing data with starting times across all days of the week. Data are the mean difference between the masked (i.e., Zeros and NaNs) and true IV values or imputed (i.e., Linear interpolation, Mean ToD, and Median ToD) and true IV values, as extracted from Bland-Altman plots. Values are presented for varied durations between bouts (y-axis) and timing (x-axis) of masked data gaps and are color-coded as indicated with best performance being closer to 0. NaN values indicate where values could not be calculated due to dataset constraints.

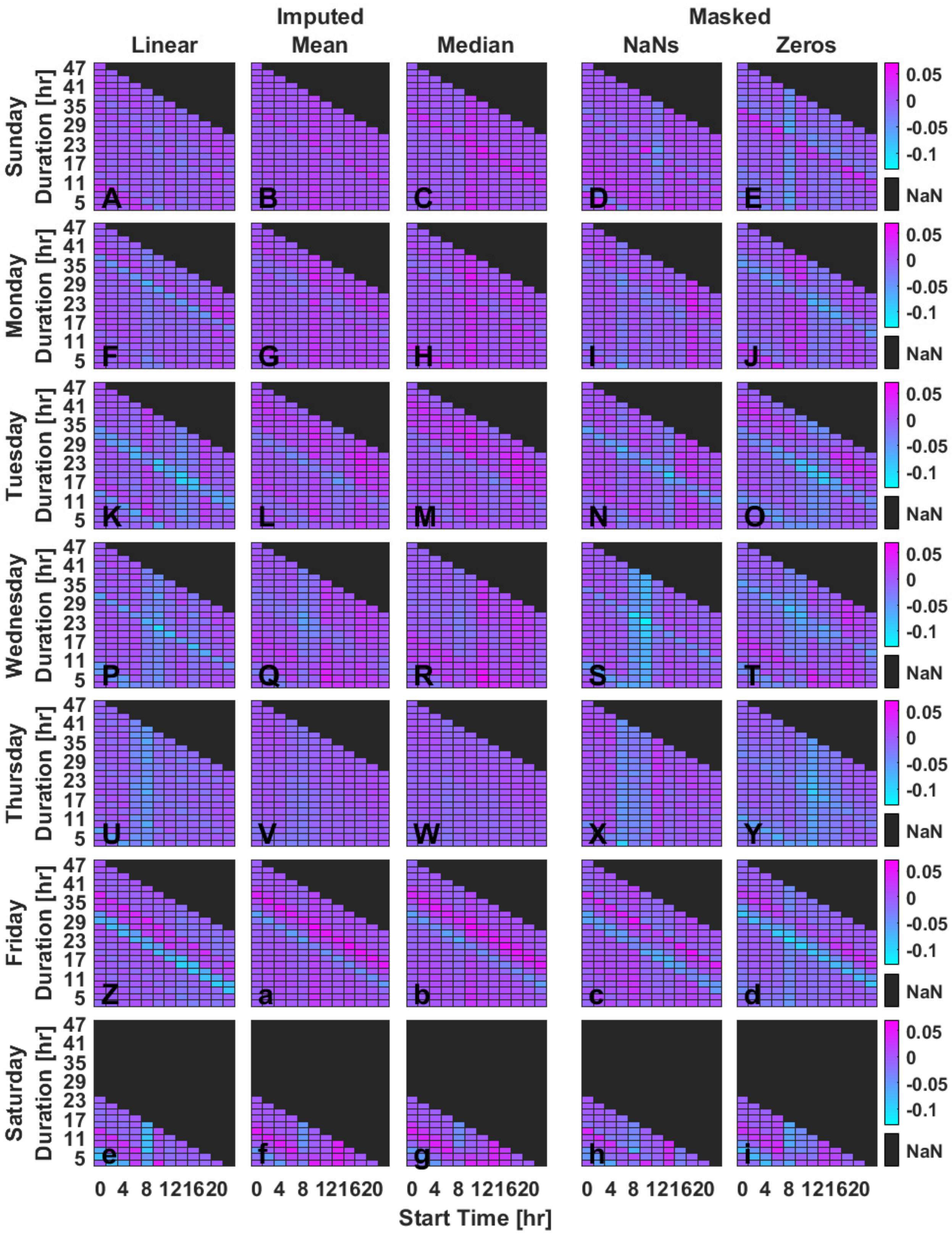

Figure S13. IV Bland-Altman slope for two gaps (gap 1: 115 minutes, gap 2: 140 minutes) of missing data with starting times across all days of the week. Data are the mean difference between the masked (i.e., Zeros and NaNs) and true IV values or imputed (i.e., Linear interpolation, Mean ToD, and Median ToD) and true IV values, as extracted from Bland-Altman plots. Values are presented for varied durations between bouts (y-axis) and timing (x-axis) of masked data gaps and are color-coded as indicated with best performance being closer to 0. NaN values indicate where values could not be calculated due to dataset constraints.
